# Azeotropes as Powerful Tool for Waste Minimization in Industry and Chemical Processes

**DOI:** 10.3390/molecules25225264

**Published:** 2020-11-12

**Authors:** Federica Valentini, Luigi Vaccaro

**Affiliations:** Laboratory of Green S.O.C., Dipartimento di Chimica, Biologia e Biotecnologie, Università degli Studi di Perugia, Via Elce di Sotto 8, 06123 Perugia, Italy; federicavalentinimail@gmail.com

**Keywords:** azeotrope, waste minimization, solvent recovery, azeotropic distillation, process design, waste valorization

## Abstract

Aiming for more sustainable chemical production requires an urgent shift towards synthetic approaches designed for waste minimization. In this context the use of azeotropes can be an effective tool for “recycling” and minimizing the large volumes of solvents, especially in aqueous mixtures, used. This review discusses the implementation of different kinds of azeotropic mixtures in relation to the environmental and economic benefits linked to their recovery and re-use. Examples of the use of azeotropes playing a role in the process performance and in the purification steps maximizing yields while minimizing waste. Where possible, the advantages reported have been highlighted by using E-factor calculations. Lastly azeotrope potentiality in waste valorization to afford value-added materials is given.

## 1. Introduction

The materials not incorporated into the final desired product and the energy required for conducting a process can be considered most trivially as the major direct origin of “waste” associated to chemical production. In addition, the possible formation of undesired isomers or byproducts also results in the generation of costly waste, that being in some cases hazardous, can limit the final application of the main product. In recent decades, many efforts have been aimed at highlighting the importance of a waste prevention approach rather than focusing on the definition and control of waste disposal techniques [1,2,3]. In the shift towards more sustainable chemical production, prevention must be the priority instead of the end-of-pipe treatment. Indeed, the Environmental Protection Agency (EPA) policy for waste minimization [4] does not consider relevant the incineration, encapsulation, or chemical oxidation procedures. Waste minimization hierarchically refers to:

- Reduction at the source: generally requiring an important process modification to reduce or eliminate the generation of waste and the use of hazardous materials.
- Recycling: consisting of contaminant removal from a useful fraction of waste to allow it to be reused.

Waste minimization is also an economical opportunity, in fact by decreasing the amount of waste generated per month, the chemical industries can be more competitive in the market: according to the EPA’s Resource Conservation and Recovery Act (RCRA) they can actually reduce waste management costs.

In the context of green chemistry, principles [5] and metrics [6,7] have been listed as guidelines and quantitative tools functional for the development of sustainable processes [7]. Among these, E-factor [8] and Process Mass Intensity (PMI) [9], equivalent but from different perspectives, focus the attention toward the optimization of the resources used by comparing the mass of materials used or of waste produced in relation to the mass of product obtained. From this comparison it emerges that the pharmaceutical industry produced the highest amount of waste [10]. Most of the waste is constituted by solvents (80–90%) [10] that are generally used in huge amounts at all stages and especially during the purification steps necessary to isolate the product in high purity. 

A careful choice of the solvent used as reaction medium in combination with an efficient recovery and reuse technique is essential in the definition of modern tools for chemical production aiming at the minimization of its environmental impact [11]. Both in the context of “recycling” and “reduction at source”, azeotropes can actively play an effective role. While the formation of azeotrope can sometimes be considered an issue that may lead to additional issues in the separation process, on the other side the peculiar properties of substances to combine together to form azeotropes may be used to develop more effective processes in chemistry and chemical engineering. 

In this contribution it will be highlighted how azeotropes can be an effective tool for waste minimization in chemical processes. In particular, it will show their role in (i) the recovery and reuse of complex mixtures of solvents, (ii) the field of process modification to prevent waste production, and (iii) the synthesis of materials based on waste valorization. 

## 2. Azeotropes for Waste Recovery and Reuse

**Figure 1:**
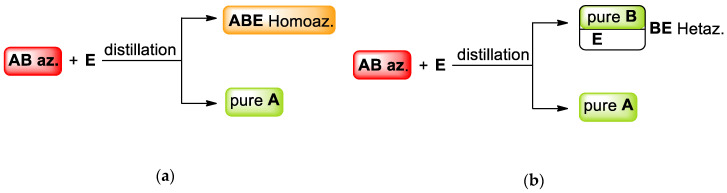
Schematic representation of (**a**) homogeneous azeotropic distillation; (**b**) heterogeneous azeotropic distillation.

By following the EPA policy for waste minimization, when the “redesign” of a process is not possible or complicated, recovery and reuse of solvent waste is crucial. As above mentioned, the formation of binary or higher-order azeotropes may constitute a problem for solvent recovery by distillation. An option could be distillation under different pressures, however, the risk of product degradation and also economic disadvantages, may lead to the definition of azeotropic distillation as an alternative effective route [12]. Azeotropic distillation (Figure 1) may also involve an entrainer (E) which changes the volatility of the components of the azeotrope forming a new azeotrope. The azeotropic distillation can be homogeneous or heterogeneous, depending if it involves a liquid−liquid separation of the new azeotrope. When the entrainer is substituted with a solvent, the distillation is named extractive distillation: the solvent interacts with one or more components and changes their fugacity without forming any new azeotrope [13]. 

**Figure 2:**
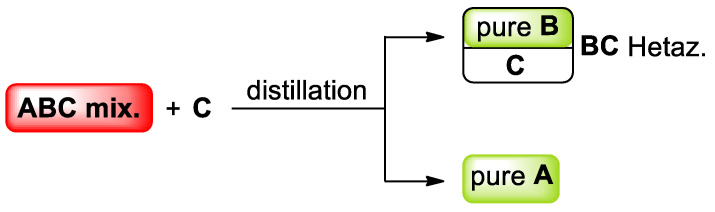
Simplified representation of extractive-heterogeneous azeotropic distillation.

By combining the extractive distillation with heterogeneous azeotropic distillation a hybrid extractive-heterogeneous azeotropic distillation (Figure 2) was proposed in 2004 [14]. One of the most important advantages of this hybrid distillation technology is that no additional solvents are added, and no new azeotropes are formed. Generally, water is present as auto-entrainer.

Below are reported significative examples on the use of azeotropic distillation for waste recovery when complex mixtures of solvents or azeotropes are present. Selected examples reflect most of the common problems of chemical industries and are complete with environmental and economic evaluations. 

### 2.1. Homogeneous Azeotropic Distillation

The first production of anhydrous ethanol with azeotropic distillation was dated 1902 and was proposed by Young [15]. The addition of benzene to an ethanol−water mixture forms a three-component azeotrope which boils at 64.85 °C. This lower boiling point, compared with the ones of ethanol−water azeotropes, allowed the distillation of the novel azeotrope formed by removing water from ethanol. In addition, the excess of benzene could be easily separated from the alcohol by using *n*-hexane. Although this method proved to be efficient and opened the way for the development of different azeotropic distillation procedures, nowadays the use of benzene is unappealing and, generally, it should be avoided. 

In this context, a practical example of waste minimization deals with the nitrocellulose manufacturing facility of Taiwan described by Liu in 2003 [16]. The waste minimization assessment plan applied at the satellite plant in Taiwan allowed for the reduction of waste at source and minimized waste, resulting in increased productivity of the facility. The alcohol rectification by azeotropic distillation was part of the recovery/recycle program. In 1978, ethanol was replaced with isopropyl alcohol (IPA) as a dehydrating agent. After concentration of the spent IPA by the two-phase column system, the formation of ternary azeotrope water−IPA−benzene allowed the recovery of up to 4 tons of IPA (99%+) daily. From 1990 to 1996 several alternatives were evaluated, and a novel rectification unit was built due to both increased production and the toxicity of benzene. The new rectification plan adopted from the task force replaced benzene with cyclohexane and the benefits from this decision allowed the recovery in two years of the USD 260,000 invested for the new rectification unit. 

In 2018, You and coworkers proposed and compared by technoeconomic analyses different azeotropic distillation designs to separate light oil waste into single components [17]. Light oil is an organic waste from Nylon plant production and is composed of *n*-pentanol, cyclohexanone and cyclohexene oxide, compounds that are difficult to separate using conventional distillation due to their similar boiling points. 

By using water as entrainer, the authors designed six different configurations for azeotropic distillation and carried out rigorous simulations, making use of the Aspen Plus software platform. The composition of the light oil was set as 35.4% of *n*-pentanol, 31.8% of cyclohexanone, and 32.8% of cyclohexene oxide in accordance with data from Yueyang Branch of Sinopec Corp. Strict parameters about purity degree of the recovered chemicals (more than 95%), their amounts (more than 92.5%) and amount of the entrainer (at least 98%) needed to be fulfilled when designing the process separation. With the results obtained from the simulations and technoeconomic analyses, it emerged that azeotropic distillation may save around 7% of the total annual costs in comparison with conventional distillation.

### 2.2. Heterogeneous Azeotropic Distillation

In cellulose acetate manufacturing, an important operation is the dehydration of acetic acid. Water and acetic acid do not form an azeotrope, and their boiling points are very similar, making conventional distillation too expensive for accomplishing the recovery of pure acetic acid. For this reason, several studies have been performed using heterogeneous azeotropic distillation [18,19]. Although these studies were focused on an optimization of the process design, an evaluation of operating costs and control of azeotropes are needed for a more comprehensive definition of a sustainable protocol. Indeed, process design and product design must be performed interactively to give a global evaluation of costs and the environmental quality of the entire process. 

In 2005 Diwekar and Xu conducted a multiobjective optimization combining process and product design to evaluate the operability of the system and the environmental impact (EI) based on LD_50_ (lethal dose for 50% of the tested population) and LC_50_ (lethal concentration for 50% of the tested population) for the continuous separation of an acetic acid−water mixture using heterogeneous azeotropic distillation [20]. The authors selected seven different environmentally benign entrainers (ethyl acetate, propyl acetate, isopropyl acetate, methyl propyl ketone, methyl isopropyl ketone, diethyl ketone, and methyl propionate) for the formation of minimum boiling point azeotropes. By considering different objectives and solvents different distillation schemes were given. The results obtained evidenced that the highest amount of recovered acetic acid was obtained by using methyl isopropyl ketone, while the best EI for LC_50_ and LD_50_ was obtained with methyl propionate and isopropyl acetate, respectively.

Chien and coworkers focused their attention on the selection of the best entrainer for acetic acid recovery in different percentages, considering the impact on total annual costs [21,22]. In 2004 the authors selected the best entrainer for the separation of an acetic acid−water mixture 1:1 [21]. Between ethyl acetate, iso-butyl acetate, and *n*-butyl acetate the authors selected the latter as best entrainer for the high ratio and purity of the acetic acid obtained. The implementation of this heterogeneous azeotropic distillation reduced the total annual cost by 55% of the value obtained for conventional distillation. Although the results obtained in this study were interesting, a typical waste stream still contained a low amount of acetic acid. For this reason, the authors in 2006 designed an alternative system for a more reasonable 8:2 water−acetic acid mixture [22]. The need for a preconcentrator column was also investigated and its impact on total annual costs evaluated. Indeed, in industrial processes, the addition of a preconcentrator column in combination with the heterogeneous azeotropic distillation is common. After careful evaluation of different parameters and configurations, the optimal design consists of a single azeotropic distillation column with aqueous reflux stream. In this configuration a decrease in total annual costs of about 25% has been reached in comparison with the preconcentrator design.

As above mentioned, the pharmaceutical industry produces the highest amount of solvent waste and a typical example of this waste stream treatment consists of the recovery of ethyl acetate-isooctane mixture. This mixture features a low boiling point azeotrope (76.3 °C). In 2014, Gerbaud and coworkers screened 13 entrainers, among 60 candidates, for the recovery of high purity compounds through heterogeneous azeotropic distillation [23]. Among these, only acetonitrile and methanol were selected as effective entrainers for heterogeneous azeotropic distillation in a rectifying column configuration. After evaluating of the miscibility gap of the heterogeneous azeotrope, the composition of the entrainer−isooctane azeotrope and the temperature differences between the other possible azeotropes, acetonitrile was selected as the best entrainer. Indeed, acetonitrile allows a decrease in operational time with an increment in yield. This study was the first example of heterogeneous azeotropic distillation for the recovery of ethyl acetate-isooctane mixture. Furthermore, the authors validated the method in a batch distillation column configuration at laboratory scale.

Another growing industry at a global scale concerns the production of semiconductors for screens. One of the major contributors to waste associated with these processes, consists of the photoresistor thinners, i.e., propylene glycol monomethyl ether (PGME) and propylene glycol monomethyl ether acetate (PGMEA). Although these components are generally retrieved by distillation, the formation of their azeotropes with water makes the recovery of these representative photoresistor thinners difficult and energy demanding. In 2016, Lee and coworkers proposed the combination of heterogeneous azeotropic distillation with a dividing wall column (DWC) [24] technology for the purification and recovery of PGME and PGMEA [25]. The authors investigated several configurations before selecting the heterogeneous azeotropic dividing wall column as the most energy and cost saving system for recovery of the photoresistor thinners. Indeed, they were able to save 33.1% of energy with an impact on the total annual costs of about 20% [25]. The energy savings in the combination of DWC technology with azeotropic distillation had already been observed, also in the dehydration of ethanol [26], bioethanol [27], acetic acid [28] and in the separation of pyridine−water−toluene mixtures [29].

### 2.3. Hybrid Extractive-Heterogeneous Azeotropic Distillation

A more complex industrial issue is related to the separation of nonideal quaternary mixtures. The waste streams studied by Mizsey in 2004 were six different nonideal mixtures composed of ethanol (EtOH), ethyl acetate (EtOAc), isopropyl acetate (IPOAc), methyl-ethyl-ketone (MEK), iso-propanol (IPOH), acetone and water (H_2_O) [14]. These solvents generate binary and ternary azeotropes and have been divided into three groups (Table 1). The authors gave general important guidelines for separating each group by using water as auto-entrainer with hybrid extractive-heterogeneous azeotropic distillation. 

Below are listed the general strategies for each group without considering dehydration of EtOH and IPOH:

- Group 1: acetone does not form any azeotropes with other components; from the economic point of view it is preferable to separate it with conventional distillation before proceeding through extractive heterogenous distillation to separate the remaining solvents by using additional water as auto-entrainer.
- Group 2: when a couple of solvents which do not form azeotropes, but their azeotropes are present with other components, extractive-heterogeneous azeotropic distillation with two subsequent conventional distillations is the preferred way to recover single components.
- Group 3: when a binary azeotrope is present between the components of the mixtures, the best way for solvent separation is two extractive-heterogeneous azeotropic distillations with subsequent conventional distillation.

This novel hybrid distillation allows the separation of complex mixtures and is a promising tool for the recovery of solvents from the waste stream with a reduced environmental impact.

To better understand the role of this new hybrid azeotropic distillation, a comparison with the conventional treatment, i.e., recovery or incineration, has been reported by the same authors in 2006 considering both environmental and economic impact [30]. A life-cycle assessment (LCA) consideration was expanded for different options of treatment of a nonideal mixture (EtOH, EtOAc, IPOAc, water) from a printing company by using Eco-indicator 99 life-cycle impact assessment methodology [31]. This method allows the assignment of a single score to the environmental impact. Focusing only on incineration and recovery by conventional distillation alternatives, economic and environmental impacts are not in agreement. Indeed, from the economic point of view, recovery is the preferable option, however, the high energy demand for separating this nonideal mixture is too high to be considered sustainable. This contradictory evidence is the driving force of green engineering to design more sustainable recovery processes. On the other hand, from these calculations, emerges the only option which leads to both economic and sustainability benefits: extractive-heterogeneous azeotropic distillation [30].

Extractive-heterogeneous azeotropic distillation is not only limited to the separation of minimum boiling point azeotropes but is useful also when those with maximum boiling points are present. Based on industrial problems, Toth and coworkers investigated the separation of two nonideal mixtures containing chloroform [32]. Chloroform generates maximum boiling point binary azeotropes both with acetone and EtOAc. After careful simulation and experimental verifications, the authors proved for the first time in 2019 the efficiency in separation of maximum boiling point azeotrope recovering chloroform at 99.5% purity.

### 2.4. Azeotropes and Membrane Technologies in Water-Containing Waste Treatment

Wastewater treatment is a complex common issue for several industries and it is also linked to the worldwide increasing demand for fresh water. Industrial wastewater comes from reverse osmosis of brine and requires the employment of membrane technologies [33,34]. Among these technologies, pervaporation is also widely employed in the removal of volatile organic compounds (VOC), dehydration of organic solvents and separation of organic mixtures [35,36,37,38,39,40]. In addition, the pervaporation approach has many advantages in the sustainable dehydration and recovery of solvents (e.g., avoids the use of entrainer, high selectivity), although a major limit can be related to the relatively high costs of membranes.

To solve this issue, hybrid technologies have been developed [41,42,43].

In 2018 a study by Andre and coworkers about the separation of an isobutanol−water mixture, emerged that the choice of a separation process strictly depends on the product composition requirement [44]. Moreover, the ranking position of a separation technology cannot be easily standardized due to social and technological factors. The authors compared different separation methods and also their combination: azeotropic distillation, azeotropic distillation extended with heat integration, pervaporation, distillation assisted pervaporation and distillation assisted pervaporation with heat integration. Multi-Criteria Decision Analysis, comprehensive of Political, Economic, Social and Technological (PEST) analysis, gave similar results to those obtained from LCA considerations. Besides considering 98.8 *w*/*w*% of isobutanol it is clear how pervaporation is the favorable choice in comparison with azeotropic distillation, by increasing to 99.9 *w*/*w*% their relative ranking position is unclear, while the hybrid combination of distillation-assisted pervaporation with heat integration is without any doubt the most convenient separation alternative in both cases. 

In 2019 similar considerations have been highlighted by Toth for the selection of the separation technology of an ethyl acetate−ethanol−water highly nonideal mixture [42]. Four different combinations of separation techniques have been economically compared considering the total annual costs (TAC). Hybrid extractive-heterogeneous azeotropic distillation allowed a reduction of the energy requirement in combination with pervaporation confirming the importance of the combination of different methodologies for saving money and energy in waste purification. Recently this combination has shown itself to be efficient also in the separation of other nonideal three-component mixtures: ethyl acetate−methanol−water and isobutyl acetate−methanol−water [43]. 

## 3. Azeotropes in Organic Synthesis for Waste Minimization

In the realm of modern organic synthesis, the strategies for minimizing the production of waste play a key role for a competitive economic and environmental chemical production and especially in the preparation of fine chemicals, modern materials or pharmaceutically active ingredients (APIs). The peculiar chemical−physical properties of azeotropes may be useful also to maximize catalyst stability and performance, for the continuous removal of byproducts, and also to influence the selectivity of the process. As above mentioned, the waste stream derived from chemical processes is mainly constituted by solvents and this is even more evident when an aqueous mixture is used as a medium as their recovery may be difficult or impossible. It is noteworthy that the use of aqueous organic mixtures (H_2_O with tetrahydrofuran (THF), dimethylformamide (DMF), ethanol (EtOH), acetonitrile (MeCN), etc.) is all but rare in routine laboratory practice but also at an industrial level. In this section it will be shown how the latter can be efficiently substituted by azeotropes both in purification steps and as a reaction medium (Section 3.3.). Below are reported relevant examples where the use of azeotropes has also led to significant improvements in the efficiency of a synthetic procedure (Section 3.1.) and or a purification process (Section 3.2.).

### 3.1. Azeotropes Steer Organic Synthesis

The use of biocatalysts for esterification processes is a very efficient process, however their facile inactivation constitutes a limitation for their use in larger scale application. In addition, considering the high costs of enzymes, preserving their stability is of fundamental importance to minimize the costs of the entire process. With this aim, Vulfson and coworkers developed a synthetic strategy to improve catalyst tolerance and allow its efficient reuse [45]. The authors found an improved catalytic activity and stability in the Novozyme 435 biocatalyst when *tert*-butanol/*n*-hexane minimum boiling point azeotrope was employed in the synthesis of sorbitan esters. The use of this azeotrope did not only allow for the reduction of the operating temperature, thus improving the preservation of the enzyme, but also for removal of the water formed during the reaction facilitating the process.

**Scheme 1:**
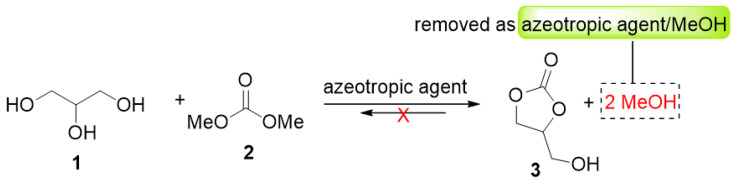
Waste-minimized synthesis of glycerol carbonate as a value-added chemical.

The use of azeotropes for the removal of byproducts, especially those that can negatively affect the course of the reaction, has been widely investigated. For example, Wang and Li reported in 2010 the use of azeotropic agents to remove methanol formed during the synthesis of glycerol carbonate (**3**) from glycerol (**1**) and dimethyl carbonate (DMC, **2**) allowing the reduction of the equivalent of the carbonylation agent [46]. Glycerol carbonate is a value-added chemical which can be produced by carbonylation of glycerol, the main byproduct of biodiesel production [47,48]. Generally, when the carbonylation of **1** is performed with DMC (**2**), an excess of the latter is required. In addition, the excess of **2** and methanol formed during the process, must be distilled for an efficient recovery of the desired product **3**. On the other hand, excess of DMC may lead to side reactions resulting in the decrease of the yield in glycerol carbonate (**3**). Low yields and an excess of reactants contribute both to the non-sustainability of the process generating waste and the requirement of additional energy costs for the recovery of the pure product. The authors proposed to exploit azeotropic distillation for the continuous remotion of methanol formed during the reaction with the aim of shifting the equilibrium of the reversible transesterification process towards the product (Scheme 1) [46]. In these conditions 98% of pure glycerol carbonate was obtained by using 1:1 ratio between glycerol and DMC. 

**Scheme 2:**
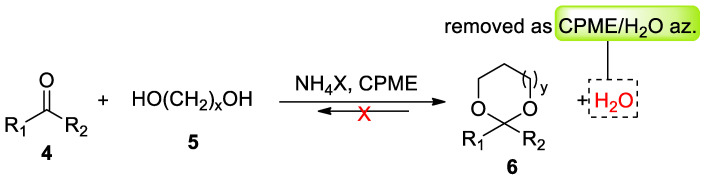
Acetalization under Dean−Stark conditions using CPME as a green alternative.

Another common example of the use of azeotropes to remove byproduct and shift the equilibrium towards product by water removal, Dean−Stark apparatus is used in the acetalization of aldehydes and ketones (**4**). In 2015, Azzena and coworkers developed a novel catalyst/solvent system as a sustainable alternative to the common toluene/*p*-toluenesulfonic acid [49]. The authors employed ammonium salts as insoluble acidic catalysts in cyclopenthyl methyl ether (CPME), a green substitute to the most common ethereal solvents, which forms a minimum boiling point heterogeneous azeotrope with water (bp 83 °C) (Scheme 2). The peculiar hydrophobicity of CPME makes this solvent almost insoluble in water, making them easy to separate and allowing an 85% recovery of the solvent. With this catalyst/solvent system they were able to perform efficient acetalization of **4** with easy recycling of ammonium salt, reducing in this way the waste production.

**Scheme 3:**
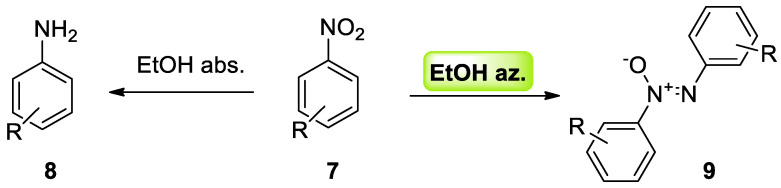
Different reaction pathways for reduction of nitroaromatic compounds promoted by EtOH azeotrope.

Azeotropes may be useful tools also in transfer hydrogenation reactions. The use of formic acid/triethyl amine azeotrope is widely employed to improve the performance of formic acid as a safer H_2_-source [50,51,52,53,54], while recently it has been observed how the azeotrope EtOH−water may shift the reaction selectivity [43]. Indeed, in 2019 Vaccaro, Piermatti and Pica realized a switchable chemo-selective Au-based heterogeneous catalyst and used this system in the reduction of nitroaromatic compounds with NaBH_4_ (Scheme 3) [55]. When absolute ethanol was used as a medium the reaction proceeded selectively towards the sole formation of the corresponding aniline (**8**). On the contrary, the employment of EtOH−water azeotrope completely shifted the selectivity with the sole formation of the azoxy derivatives (**9**) giving the possibility to isolate these important chemicals in high yields using a single catalytic system. 

### 3.2. Azeotropes in Purification Process

**Scheme 4:**
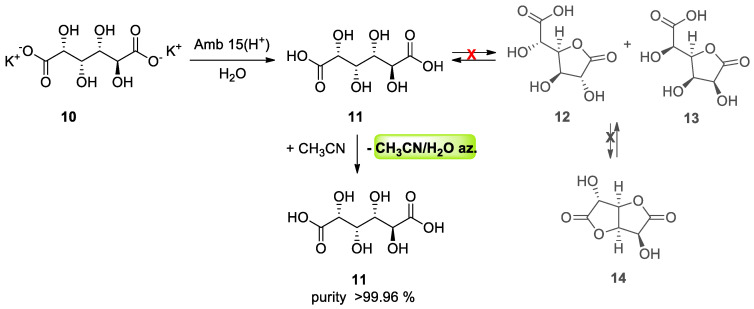
Purification of d-glucaric acid by azeotropical removal of water.

Glucaric acid is a bioderived platform molecule derived from the catalytic oxidation of glucose. Despite the great interest for this chemical as a key ingredient for several industrial specialties, it is commercialized only as K and Ca glucarate salts. Reproducible procedures for the obtainment of the pure crystalline form of d-glucaric acid (**11**) are extremely rare due to the equilibrium with the corresponding lactones (**12, 13, 14**). The dehydration leading to lactones is further promoted by high temperatures, generally employed for water removal. For this purpose, Armstrong and coworkers developed an easy method to obtain pure crystalline **11** exploiting minimum boiling point azeotrope acetonitrile−water [56]. Starting from commercially available glucarate salts (**10**) the authors employed Amberlyst-15 (H^+^) exchange resin to promote the formation of **11**, when the addition of acetonitrile allows the drying of the product at a lower temperature without any formation of byproducts or additional gains in energy demand (Scheme 4). With this set-up the authors were able to isolate up to 98% of pure dry d-glucaric acid (>99.96%). The reproducibility of this method leads to further study of life-cycle assessment and technoeconomic analysis for the preparation of pure crystalline d-glucaric acid starting from glucose oxidation [57]. Efficient strategies for high yield product isolation are, indeed, powerful and sustainable tools for waste minimization.

**Scheme 5:**
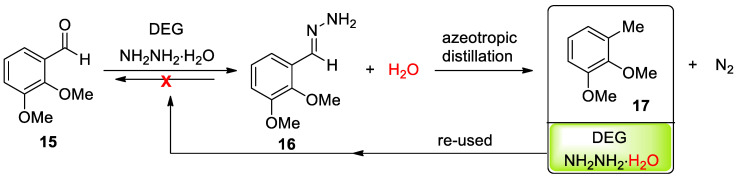
Continuous Wolff−Kishner reduction recovering and reusing hydrazine hydrate and diethylene glycol (DEG) by heterogeneous azeotropic distillation of product **17**.

Eisenbraun and coworkers developed an efficient method for Wolff−Kishner reduction employing diethylene glycol (DEG) which forms bilayer azeotropes with products [58]. The Wolff−Kishner reduction allows for the obtainment of methylene groups from carbonylic functionalities making this reaction extremely useful in organic chemistry and several modifications have been largely applied over the years. Glycols were generally employed to allow an increase in the reaction temperature and facilitate the removal of water [59,60,61] formed during the reaction, and also removal of excess of hydrazine hydrate [61]. However, due to the extreme conditions of these conditions, further improvements were desirable. Eisenbraun and coworkers therefore proposed an easy way to perform the Wolff-Kishner reduction employing azeotropic distillation with the preservation of KOH, recycling the excess hydrazine hydrate and a portion of DEG (Scheme 5) [58]. The authors found that products (**17**) from dimethoxybenzaldehyde (**15**) and 2′-acetonaphthone form a heterogeneous azeotrope with diethylene glycol and hydrazine hydrate. This consideration allows the continuous separation of products from the reaction mixture and recover DEG and hydrazine hydrate. The possibility to preserve KOH and reuse hydrazine hydrate for several consecutive runs, in combination with the 85% of DEG recovered, make this process a sustainable alternative which minimizes waste by reducing reactant costs and reaction time as well. 

### 3.3. Azeotropes Enable Waste Prevention

**Scheme 6:**
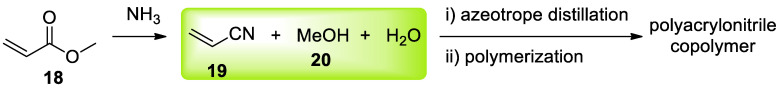
Polyacrylonitrile polymerization by azeotropic distillation of **19** obtained from nitrilation of **18**.

The ever-increasing interest in the carbon fiber market is due to the peculiar properties of these materials and broad applicability range [62]. Among different precursors, polyacrylonitrile is the most employed, however a key limitation factor for its application is related to the high cost of its key-ingredient, acrylonitrile. Its cost is strictly related to the synthetic strategies used (propylene ammoxidation), which also lead to the formation of several byproducts [63,64,65]. The need for a more sustainable synthesis, preferably based on renewable feedstock, is steering the research towards a biomass-based production of acrylonitrile (**19**) [66,67]. Recently, Naskar, Beckham and coworkers proposed an integrated synthesis with subsequent polymerization without the need for any additional acrylonitrile isolation/purification [68]. The authors used the acrylonitrile synthesized by nitrilation of methyl acrylate (**18**) directly in the polymerization step (Scheme 6), minimizing energy and material costs for acrylonitrile purification. Acrylonitrile forms a ternary azeotrope with water and methanol (MeOH) **20**, therefore it separates and can be used in emulsion polymerization with MeOH as chain transfer agent. This set-up has several environmental advantages, such as reduced operational costs avoiding purification, minimized energy demand and substitution of conventional thiol-based chain transfer agents with MeOH as a coproduct (**20**) formed in the nitrilation step. The authors calculated that the use of **19**−**20**−water/azeotrope impacts on the total process for about 40% of the imported electricity and 35% of the heat demand. This waste-minimized method for the production of polyacrylonitrile copolymer showed comparable efficiency with conventional methods allowing high molecular weight and low polydispersity of the obtained material. 

Particularly intriguing is the direct utilization of aqueous azeotropes as the reaction medium for process modification aimed at waste minimization [55,69,70,71,72,73,74,75].

**Scheme 7:**
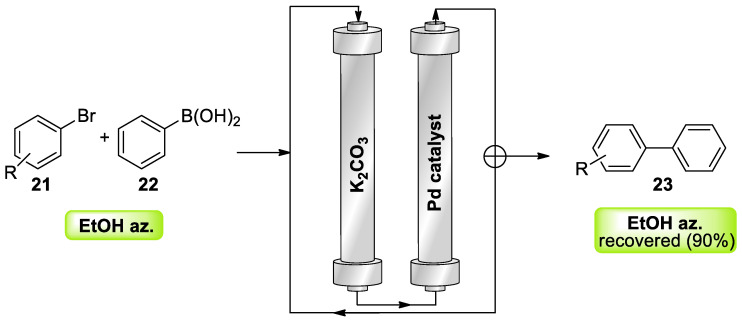
Flow approach for the waste-minimized Suzuki reaction in aqueous EtOH azeotrope.

In 2013 a silica-base heterogeneous catalyst was employed in Suzuki cross-coupling and the role of ratio of EtOH:water mixture in the catalytic performance has been investigated [69]. The authors found the best reaction conditions in batch the employment of the EtOH:water mixture in 1:1 ratio. This medium selection is based on the search for the complete dissolution of both organic and inorganic reagents or additives. Anyway, comparing aqueous EtOH azeotrope with absolute EtOH, yield of the product increases significantly. Despite the heterogeneous catalyst showing good recyclability under the optimized batch conditions, the authors aimed to further minimize the environmental impact of the Suzuki cross-coupling by employing flow technology (Scheme 7).

The flow set-up was designed with the intention of exploiting the well-established “release and catch” mechanism of Pd-catalysts [76,77]. Two different reactors were separately filled with K_2_CO_3_ base and Pd-based catalyst; the mixture of reactants in EtOH aqueous azeotrope was cyclically pumped until complete conversion was achieved. The use of azeotrope was found to be essential for the strategy, in fact, K_2_CO_3_ is insoluble in this medium and therefore acts as a heterogeneous system able to scavenge the boronic acid formed during the reaction allowing the product to be isolated in pure form without an additional purification step. Solid base therefore can be charged into a reactor separated from the catalyst allowing easy preservation of the efficiency of the catalytic system and easy recovery of it. Product is isolated by simple distillation of EtOH azeotrope that can be recovered and resulting in E-factor values between 3.5–3.9. Common E-factor values calculated for previously reported protocols were in the range of 3180−5100 due to the utilization of mixtures of solvents used for running the reaction, for the work-up procedure and the required purification steps. The combination of flow technology [78] and azeotrope medium significantly decrease waste production for the Pd-catalyzed Suzuki cross-coupling allowing a 99% E-factor minimization [69]. 

A similar protocol was employed by Vaccaro and coworkers using zirconium phosphate glycine diphosphonate nanosheets as support for Pd nanoparticles for Suzuki cross-coupling reaction [70]. The use of EtOH aqueous azeotrope was found to be essential for leaching minimization and preserving the catalyst for several consecutive runs. Simple distillation of the azeotropic reaction media allowed pure crystalline products to be obtained with an E-factor value of 3.2.

**Scheme 8:**
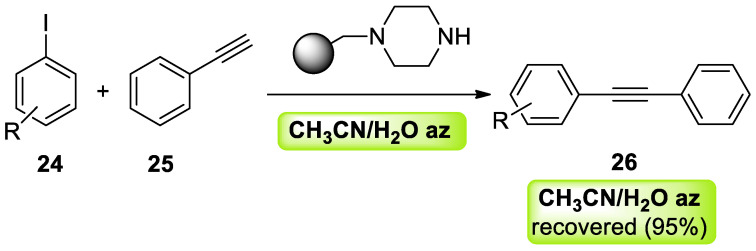
Sonogashira reaction in aqueous acetonitrile azeotrope.

Generally, cross-coupling reactions are performed in toxic dipolar aprotic solvents such as DMF (dimethylformamide) and NMP (*N*-methyl-2-pyrrolidone), the employment of these solvents, often coupled with water to dissolve inorganic salts, reflects an unsustainable waste generation for their complete removal from products. Among solvents which can be used as substitutes for DMF and NMP, acetonitrile is of great interest due to the possibility of forming a minimum boiling point azeotrope with water. Vaccaro and coworkers employed a synthetic strategy for waste minimization of Sonogashira cross-coupling (Scheme 8) by using recoverable aqueous acetonitrile azeotrope as the reaction medium and a heterogeneous recoverable catalyst/base system [71]. 

The synthetic strategy reported [71] achieves E-factor values from 8 to 20 with an 82% waste minimization if compared with the protocol that uses a homogeneous base. The use of polymer-supported piperazine base in combination with acetonitrile azeotrope significatively decreases Pd leaching, maximizing the recovery and reuse of the catalyst. Indeed, it is well known that DMF and NMP may stabilize metal released in solution leading to a tedious procedure for product purification.

**Scheme 9:**
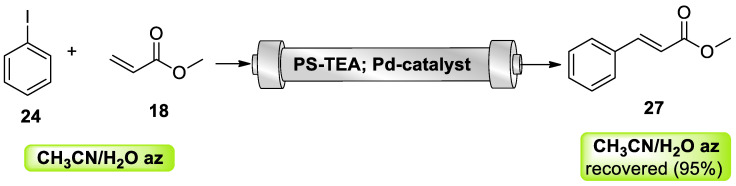
Mizoroki−Heck cross coupling in aqueous acetonitrile azeotrope.

Due to the synthetic importance of the Mizoroki−Heck cross-coupling reaction in pharmaceutical and opto-electronic industries, two waste-minimized flow protocols have been developed in acetonitrile/water azeotrope with different Pd-heterogeneous catalysts using polymer supported triethylamine as the base (Scheme 9) [72,73]. The flow reactor was packed with a dispersion of heterogeneous base and Pd-catalyst and then a mixture of iodobenzene (**24**) and methyl acrylate (**18**) in acetonitrile water azeotrope was pumped at 130 °C. When potassium α-zirconium phosphate was employed as support for Pd nanoparticles [73] an E-factor value of 4.6 was obtained, while slightly higher values (7–12) were measured with silica-supported ionic liquid for Pd stabilization was employed as support [72]. In both cases the azeotrope acetonitrile water was recovered to 95%, allowing pure products to be obtained without the need of additional steps. The developed protocol highlighted the performance of azeotrope in waste prevention: all the input materials have been recovered or efficiently transformed minimizing the environmental impact of this useful tool for C–C bond formation.

Most important for an effective sustainable approach, the use of renewable feedstock is essential to reduce the carbon footprint of the chemical synthesis. Indeed, the replacement of fossil-derived with biomass-derived solvents plays a crucial role in the design of more sustainable synthesis [79,80]. 

**Scheme 10:**
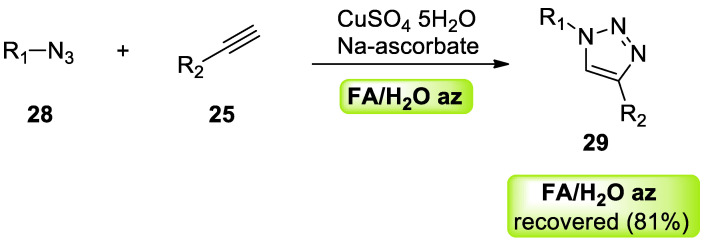
Copper-catalyzed azide–alkyne cycloaddition in recoverable furfuryl alcohol water azeotrope as reaction medium.

Furfuryl alcohol is a clear liquid with high boiling point (171 °C) derived from the treatment of hemicellulose [81]. This biomass-derived chemical is miscible with several organic solvents and forms a minimum boiling point azeotrope with water (bp 98.5 °C). For the first time in 2016 the furfuryl alcohol aqueous azeotrope was used in copper-catalyzed azide–alkyne cycloaddition (Scheme 10) by Vaccaro and coworkers [74]. The authors proved the efficiency of this reaction medium obtaining good yields of product with different substrates. In addition, the presence of a portion of water in the azeotrope allowed the product to precipitate making the purification procedure easier. Indeed, simple filtration was necessary to obtain pure products (**29**) and 81% of initial azeotrope was recovered and reused for several consecutive runs without any loss in efficiency. 

This improved synthetic strategy allows a decrease of 96% of the waste generated for this process resulting in an E-factor value of 4.3. The use of furfuryl alcohol water azeotrope is environmentally advantageous not only because of its easy recovery and reuse but also for the life-cycle assessment consideration about the employment of biomass derived solvents. 

**Scheme 11:**
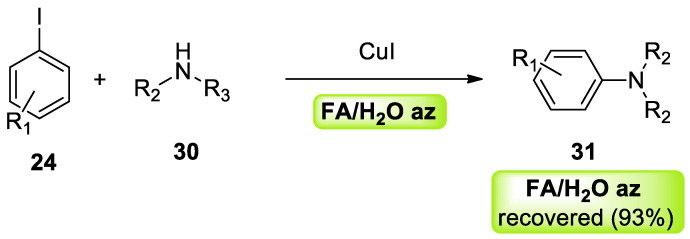
Ullman-type cross-coupling in recoverable furfuryl alcohol water azeotrope.

The furfuryl alcohol chemical structure make its use interesting also as a bidentate ligand for metal complexes. In 2018 Vaccaro and coworkers tested the efficiency of furfuryl alcohol and other oxygen-containing bidentate ligands in copper-catalyzed Ullman-type cross-coupling reactions (Scheme 11) [75]. The best reaction conditions for the coupling of iodoarene derivates (**24**) with heteroaromatic or aliphatic amine (**30**) were obtained with furfuryl alcohol and tetrahydrofurfuryl alcohol as ligands. Due to the possibility of forming aqueous azeotropes with water, the authors chose to perform the Ullman coupling reaction in furfuryl alcohol azeotrope. In addition, several experiments were conducted to evaluate the influence of furfuryl alcohol by changing its ratio with water. Optimal results in terms of conversion were obtained only by using the azeotropic mixture to efficiently synthetize 23 different products with low environmental impact. The authors were able to recover 93% of furfuryl alcohol azeotrope minimizing E-factor values to 9.7. This constitutes the first example in which both reaction medium and ligand could be easily recovered and reused, minimizing waste generation.

## 4. Azeotropes in Material Synthesis for Waste Valorization

When waste minimization is not possible, a powerful tool for minimizing the environmental impact of chemistry is the ability to transform waste stream into value-added materials. In this field azeotropes may also play a crucial role. Below are reported two significant examples of waste valorization driven by azeotropes. 

**Scheme 12:**
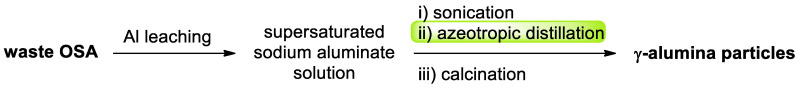
Waste OSA valorization to γ-alumina particles.

Fly ash derived from oil shale processing is a serious environmental pollutant. Gan and coworkers reported in 2010 the use of oil shale ash (OSA) as alumina source in the preparation of gamma-alumina nano-powder (Scheme 12) [82]. Alumina nanoparticles found applications in different fields from catalysis to ceramics as well as their use as absorbents. The authors combined ultrasound technology with azeotropic distillation to afford good size distribution of γ-alumina. Indeed, sonication promoted the contact between *n*-butanol and hydrogel preventing aggregate formation, while azeotropic distillation efficiently removed water adsorbed on particle surfaces. This work represents a valid alternative for OSA valorization.

**Scheme 13:**
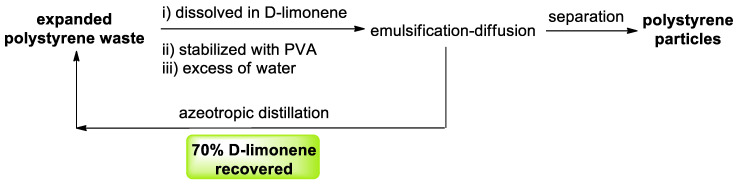
Synthesis of value-added polystyrene particles from expanded polystyrene waste and solvent recovery through azeotropic distillation.

Expanded polystyrene is a non-biodegradable material widely employed in packaging that constitutes a serious environmental issue. Among the two alternatives for expanded polystyrene recycling are energy recovery by incineration and valorization by its use as a raw material; the latter is usually expensive while incineration may generate hazardous chemicals. In 2016 Varughese and Mangalara developed a sustainable approach for synthetized value-added polystyrene particles with micro- or nano-size from expanded polystyrene waste through an emulsification−diffusion sequence (Scheme 13) [83]. The authors employed bioderived d-limonene to dissolve polystyrene and performed emulsification in aqueous medium, after adding an excess of water to promote the precipitation of new polystyrene particles (diffusion), the recovery of d-limonene was performed at a relatively low temperature thanks to the formation of d-limonene water heterogeneous azeotrope (bp 97.4 °C). The yield and size effect of the polystyrene particles were found to be dependent on the amount of poly(vinyl alcohol), employed as a biodegradable emulsification stabilizer. Up to 70% of d-limonene employed for the expanded polystyrene valorization was efficiently recovered making this novel approach a sustainable alternative to the conventional mechanical recycle and incineration.

## 5. Conclusions and Future Outlook

In the shift towards more sustainable chemical production several efforts have been made to develop synthetic technologies aimed at waste minimization. In this review we have tried to highlight and discuss the possible role of azeotropes both in waste recovery and reuse, as alternatives to the more employed end-of-pipe treatments: incineration, and in process modification. Lastly, a few examples of their use in waste valorization have been reported. 

Significant examples in the use of azeotropic distillation for solvent recovery from the waste stream have been discussed, highlighting both the environmental and economic gains considering homogeneous azeotropic distillation, heterogeneous azeotropic distillation and hybrid extractive-heterogeneous azeotropic distillation. 

More importantly, by following EPA hierarchy, the process modification design for “source reduction” is a fundamental strategy. In this context it has been highlighted the importance of azeotropes to assist organic synthesis by preserving catalysts or in the purification processes for the isolation of pure product by minimizing waste and energy or also with their use as recoverable reaction media. 

In addition, examples of the use of azeotropes for the implementation of a sustainable approach to waste valorization have been discussed.

Despite azeotropes proving to be a powerful tool for waste minimization during the different synthetic steps, there are a small number of examples in literature that consider the carbon footprint and LCA of the solvents employed. In these optics, azeotropes of biomass-derived chemicals should be further employed to increase the sustainability of known, less environmentally effective protocols.

## Figures and Tables

**Table 1:** Groups of nonideal mixtures with number of azeotropes formed.

	Mixture	n. Binary Azeotropes	n. Ternary Azeotropes
Group 1	Water, EtOH, MEK, acetone	3	1
	Water, EtOH, EtOAc, acetone	3	1
Group 2	Water, EtOH, EtOAc, IPOAc	5	2
	Water, EtOH, MEK, IPOAc	5	2
Group 3	Water, EtOH, EtOAc, MEK	6	3
	Water, IPOH, EtOAc, MEK	6	3
